# Functional Connectivity of Chronic Cocaine Use Reveals Progressive Neuroadaptations in Neocortical, Striatal, and Limbic Networks

**DOI:** 10.1523/ENEURO.0081-18.2018

**Published:** 2018-07-24

**Authors:** Caitlin A. Orsini, Luis M. Colon-Perez, Sara C. Heshmati, Barry Setlow, Marcelo Febo

**Affiliations:** 1 Department of Psychiatry, University of Florida, Gainesville, FL 32611; 2 Department of Neuroscience, University of Florida, Gainesville, FL 32611; 3 Department of Psychology, University of Florida, Gainesville, FL 32611; 4 Center for Addiction Research and Education (CARE), University of Florida, Gainesville, FL 32611; 5 Advanced Magnetic Resonance Imaging and Spectroscopy (AMRIS) Facility, University of Florida, Gainesville, FL 32611

**Keywords:** cocaine, functional connectivity, network analysis, rat, resting-state fMRI, self-administration

## Abstract

Brain imaging studies indicate that chronic cocaine users display altered functional connectivity between prefrontal cortical, thalamic, striatal, and limbic regions; however, the use of cross-sectional designs in these studies precludes measuring baseline brain activity prior to cocaine use. Animal studies can circumvent this limitation by comparing functional connectivity between baseline and various time points after chronic cocaine use. In the present study, adult male Long–Evans rats were trained to self-administer cocaine intravenously for 6 h sessions daily over 14 consecutive days. Two additional groups serving as controls underwent sucrose self-administration or exposure to the test chambers alone. Functional magnetic resonance imaging was conducted before self-administration and after 1 and 14 d of abstinence (1d and 14d Abs). After 1d Abs from cocaine, there were increased clustering coefficients in brain areas involved in reward seeking, learning, memory, and autonomic and affective processing, including amygdala, hypothalamus, striatum, hippocampus, and thalamus. Similar changes in clustering coefficient after 1d Abs from sucrose were evident in predominantly thalamic brain regions. Notably, there were no changes in strength of functional connectivity at 1 or 14 d after either cocaine or sucrose self-administration. The results suggest that cocaine and sucrose can change the arrangement of functional connectivity of brain regions involved in cognition and emotion, but that these changes dissipate across the early stages of abstinence. The study also emphasizes the importance of including baseline measures in longitudinal functional neuroimaging designs seeking to assess functional connectivity in the context of substance use.

## Significance Statement

Although human neuroimaging studies have been invaluable in understanding the relationship between cocaine use and brain functional connectivity, they inherently lack predrug baseline information that is important to establish how cocaine use alters regional interactions. Coupling neuroimaging with rodent models of cocaine self-administration circumvents this issue by controlling variables that can confound human studies and providing predrug baseline information. Using such an approach, this study reveals that after cocaine use, functional connectivity patterns change as a function of abstinence duration and that these changes are observed in networks supporting reward and emotion-related cognition and behavior. These findings highlight the importance of using multiple time points in preclinical models of substance use to assess effects on functional connectivity.

## Introduction

The use of illicit substances continues to represent a major health and socioeconomic challenge affecting the lives of many in the United States and worldwide. In 2013, >24.5 million individuals in the United States reported the use of illicit substances, and of those, 1.5 million reported use of the psychostimulant cocaine (Lipari et al., 2013). The extent and severity of substance use therefore warrants more preclinical research to uncover the neural bases of this condition and develop targeted diagnostic and treatment strategies. Decades of research have shown that the mesocorticolimbic system is unequivocally involved in reward processing and motivated behavior and is altered by chronic cocaine use (Volkow and Morales, 2015). These alterations include changes in synaptic dopamine (DA) and glutamate release and uptake homeostasis (Kalivas et al., 2009; Volkow and Morales, 2015), and changes in synaptic plasticity in the nucleus accumbens (NAc) and prefrontal cortex (PFC; Kolb et al., 2003; Kauer and Malenka, 2007). Such changes, although at the molecular and cellular level, have the potential to profoundly modify how activity in these brain reward regions functionally interacts within larger-scale neural networks.

Human neuroimaging studies have provided evidence for significant changes in neural activity across brain regions in cocaine users (Li et al., 2000; Gu et al., 2010; Ma et al., 2010, 2011; Kelly et al., 2011; Cisler et al., 2013; Konova et al., 2013; McHugh et al., 2013, 2014; Camchong et al., 2014; Hu et al., 2015). For instance, using functional magnetic resonance imaging (fMRI), cocaine-dependent subjects showed reduced resting-state functional connectivity among PFC, amygdala, and hippocampus, and between the ventral tegmental area and an area comprising the lentiform nucleus and putamen, which correlated with years of cocaine use (Gu et al., 2010). In abstinent cocaine users, there is reduced interhemispheric functional connectivity in lateral PFC, medial premotor, and lateral parietal cortices (Kelly et al., 2011), and in relapsed substance users, reduced connectivity between the corticomedial amygdala and ventromedial and rostral anterior cingulate cortices (McHugh et al., 2014). Furthermore, a recent study demonstrated that reduced resting-state functional connectivity between the NAc and PFC regions in individuals in whom substance use disorders have been diagnosed is associated with worse performance on laboratory measures of cognitive control, suggesting that changes in neuronal activity between these regions underlie cognitive and behavioral deficits in chronic cocaine users.

While these studies show that reduced functional connectivity across brain regions is associated with cocaine use, the brain regions involved and the extent of the connectivity changes among them depend on several factors. For example, years of cocaine use, current or recent cocaine use, duration of abstinence (Abs), relapse status, treatment history, and comorbid conditions all may determine connectivity changes within mesocorticolimbic networks, as well as the magnitude of these changes. The specificity of these effects is unclear, however, because of the inherent difficulties in human imaging studies in cocaine users. In addition, such studies preclude the determination of whether changes in connectivity are a result of cocaine use or whether they represent a pre-existing vulnerability (which could ideally be assessed by including a baseline, cocaine-naive measurement). Preclinical models of cocaine use can therefore be useful as they allow for controlled determination of the conditions in which chronic cocaine use impacts the intrinsic functional connectivity of the brain. To date, only a few preclinical studies (Gozzi et al., 2011; Lu et al., 2014) have assessed cocaine-induced alterations in mesocorticolimbic and corticostriatal connectivity. These studies, however, did not include a baseline measure against which to compare connectivity following cocaine use. To this end, in the current study, functional magnetic resonance imaging was used both before and at two time points after intravenous cocaine self-administration (SA) in rats to assess how cocaine use alters brain functional connectivity.

## Materials and Methods

### Subjects

Male Long–Evans rats (*n* = 21; 60 d old; Charles River Laboratories) were individually housed and kept on a 12 h light/dark cycle (lights on at 6:00 P.M.) with free access to water and food, except as noted below. Before imaging procedures, rats were handled two to three times to habituate them to the researchers. During the 3 weeks of shaping and cocaine or sucrose self-administration, rats were limited to 30 g of food per day (and water *ad libitum*) to minimize the effect of motivational differences due to natural variations in food intake and weight gain across rats. Additionally, prior work (Carroll et al., 1979) has shown that food restriction augments cocaine self-administration relative to an *ad libitum* diet. Behavioral procedures were conducted between 9:00 A.M. and 6:00 P.M., 7 d/week. All animal procedures were approved by the University of Florida Institutional Animal Care and Use Committee and followed NIH guidelines.

### Self-administration apparatus

Self-administration procedures were conducted in 12 identical standard rat behavioral test chambers (30.5 × 25.4 × 30.5 cm; Coulbourn Instruments) housed in sound-attenuating cubicles. Each chamber was equipped with two nose-poke holes located on the left and right side of the front wall, which could be illuminated by lights located inside the holes. Twenty milliliter syringes mounted on infusion pumps (Coulbourn Instruments) were used for intravenous drug delivery to rats in each test chamber. The syringes were connected to a tether system (Instech Laboratories) consisting of PE50 tubing that ran from the syringe to a fluid swivel and from there to a fluid line that mated to the back-mounted venous access port. Each chamber was equipped with a liquid dipper trough located in the center of the front wall for delivery of sucrose solution. The chambers were interfaced with a computer running Graphic State 3.0 software to control drug delivery and record nose-poke data from each of the chambers. During self-administration sessions, only one of the two nose-poke holes (the “active” hole) was illuminated (the left/right position of the illuminated hole was counterbalanced across rats and groups and remained constant across all sessions).

### General experimental design

**Figure 1.**
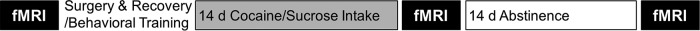
Experimental timeline showing each of the imaging sessions in relation to periods of cocaine or sucrose self-administration and abstinence.

After a week of acclimation to the vivarium and handling, rats underwent the first imaging session to assess baseline functional connectivity (imaging and image-processing methods are provided below). All rats then underwent jugular catheter surgery [regardless of whether they were in the SA (cocaine or sucrose) or chamber exposure control conditions]. The first imaging session took place before surgery, to reduce the possibility of potentially confounding contributions of a systemic inflammatory response during the period of surgical recovery (Dipasquale et al., 2016; Marsland et al., 2017). This design also allowed self-administration to commence immediately after surgery, increasing the likelihood of patent catheters, which become less likely as the time between surgery and self-administration increases. Both cocaine and sucrose self-administration groups were shaped to perform the various components of self-administration behavior (e.g., nose poking in the active hole for sucrose), after which they were then trained to self-administer cocaine or sucrose for 14 d (details provided below). Rats in the chamber exposure control group were tethered in the test chambers but did not self-administer cocaine or sucrose (i.e., the nose-poke holes were not active). One day after the last self-administration or exposure control session, rats underwent a second imaging session and were then left undisturbed in their home cages (Fig. 1, timeline). Rats then underwent a third and final imaging session 14 d after the last self-administration session, a time point at which previous studies have observed both neurobiological and behavioral changes relative to day 1 of abstinence from cocaine self-administration (Doyle et al., 2014; Glynn et al., 2018).

### Surgery

Rats were anesthetized with isoflurane gas (1–5% in O_2_) and administered Metacam (1 mg/kg), buprenorphine (0.05 mg/kg) and sterile saline (10 ml) subcutaneously. Using aseptic surgical techniques, the top of the right jugular vein was ligated and a catheter (Instech Laboratories) was inserted into the middle of the vein and sutured into place. The other end of the catheter was passed subcutaneously over the right shoulder and through a small incision in the skin over the scapulae. The end of the catheter was then attached to a back mounted port (Instech Laboratories). After the fascia of the back skin was removed, the port was nestled underneath the skin and sutured into the surrounding muscle. The skin around the back port was then sutured and a protective aluminum cap was placed on the port. Rats were given 5 d to recover from surgery, after which they were food restricted and began shaping for cocaine or sucrose self-administration. Catheters were flushed daily with heparinized saline and checked weekly for patency with an intravenous infusion of 0.1 ml of propofol, which results in rapid but transient loss of muscle tone.

### Self-administration procedures

Self-administration began with rats learning to perform the basic components of the task. Following magazine training, during which rats learned to enter the liquid trough to obtain 40 µl of a 20% sucrose solution, rats were trained to nose poke in the active hole to activate the liquid dipper to access the sucrose solution. After reaching a criterion of 50 nose pokes in 30 min (which took three to four sessions), the rats were moved on to the full self-administration sessions. For rats in the cocaine group, nose pokes into the active nose-poke hole were reinforced on a fixed ratio 1 (FR1) schedule by delivery of cocaine HCl (dissolved in 0.9% sterile saline, 1.0 mg/kg/infusion; Drug Supply Program, National Institute on Drug Abuse) in a volume of 0.16 ml over 6 s, followed by a 20 s timeout period. Nose pokes at the nonilluminated (inactive) nose-poke hole were recorded but had no programmed consequences. Cocaine self-administration sessions lasted for 6 h/d for 14 d.

Rats in the sucrose group were trained to nose poke on an FR1 schedule to obtain access to the sucrose solution via the liquid dipper on a schedule such that the number of sucrose reinforcers allowed to be earned by each rat was matched to the number earned by a partnered cocaine rat for each self-administration session (e.g., if a cocaine rat earned 30 cocaine infusions in a session, its sucrose partner was allowed to self-administer only 30 sucrose deliveries in its session on that day). This procedure was designed to equate instrumental experience and the number of reinforcer deliveries across the two groups for each of the 14 d of training (Mitchell et al., 2014a,b). Rats in the chamber exposure control group were tethered in the test chamber, but, unlike the cocaine and sucrose groups, they did not have access to the sucrose solution or cocaine and were left undisturbed for 3 h each day for 18 consecutive days (to match the average number of days in the chamber for the self-administration groups). The duration of time spent in the chambers for this control group was chosen because it was approximately the midpoint of the time spent in the chambers between the cocaine (6 h) and sucrose (∼1–2 h, depending on the cocaine intake of the partnered cocaine rats) groups.

### Functional magnetic resonance imaging

Rats were imaged under combined dexmedetomidine (0.02 mg/kg) and isoflurane (0.5%) anesthesia, as previously published (Colon-Perez et al., 2016). The spontaneous breathing rate was monitored during setup and MRI acquisition using monitoring and gating apparatus (SA Instruments). Body temperature was maintained at 37–38°C using a warm water recirculation system (Gaymar). Images were collected on a 4.7 T/33 cm horizontal magnet (Magnex Scientific) with an 11.5-cm-diameter gradient insert (670 mT/m maximum gradient strength at 300 A and a 120 µs rise time; Resonance Research) and controlled by VnmrJ 3.1 software (Agilent). A quadrature transmit/receive radio frequency (RF) coil tuned to 200.6 MHz ^1^H resonance was used for B_1_ field excitation and RF signal detection (Air MRI). Functional images were collected using a two-shot spin-echo echoplanar imaging (EPI) sequence with the following parameters: echo time (TE) = 50 ms; repetition time (TR) = 1 s; 32.5 × 32.5 mm in plane; 12 slices with 1.5 mm thickness per slice; data matrix = 64 × 64. A total of 210 repetitions were collected per EPI scan (7 min), with two scans per rat. No stimuli were presented during functional scanning. Anatomic scans for image overlay and reference-to-atlas registration were collected using a fast spin echo sequence (TE = 45 ms; TR = 2 s; echo train length = 8; number of averages = 10; data matrix = 256 × 256) in the same space as the EPI scan.

### Resting-state image processing

Resting-state functional MRI was used to assess correlations in intrinsic neural activity in the rat brain. The BOLD signal has previously been shown to have a closer relationship with somatodendritic field potentials than with neuronal spiking activity (Logothetis et al., 2001). Functional connectivity, as implemented in the present study, is considered to reflect a combination of vascular, hemodynamic, and somatodendritic activity that correlates across specific pairwise combinations of regions of interest (ROIs). Thus, the term “connectivity” is used interchangeably with correlated neural activity, which may be shared between structures having direct axonal projections or between regions without first-order or even second-order axonal connections (Adachi et al., 2012; O’Reilly et al., 2013).

Brain masks were manually generated using high-resolution anatomic scans with the help of itk-SNAP (www.itksnap.org). The masks outlining the brain were used to remove nonbrain voxels. The cropped brain images were aligned with a rat brain template using the FMRIB Software Library linear registration program *FLIRT* (Jenkinson et al., 2002). Registration matrices for each subject were saved and used to subsequently transform functional datasets into atlas space for preprocessing and analysis. Slight displacements in individual images over the series of 210 images and slice timing delays were corrected, and time series spikes were removed using Analysis of Functional NeuroImages (AFNI; Cox, 1996). Linear and quadratic detrending, spatial blurring (1.1 mm FWHM), and intensity normalization were performed. Head-motion parameters and cerebroventricular and white matter signals were extracted based on their location in the segmented atlas and were removed from datasets. A voxelwise temporal bandpass filter (between 0.01 and 0.1 Hz) was applied before time series correlation analyses to remove brain signals that contain higher-frequency oscillations.

Time series fMRI signals were extracted from each ROI based on the atlas-guided seed location (75 bilateral placed seed regions included for 150 total ROIs). Time series for each voxel were averaged per ROI seed, and voxelwise cross-correlations were conducted to create correlation coefficient (Pearson *r*) maps (Colon-Perez et al., 2016). The first nine images in each functional time series were not used in the cross-correlation step to avoid including unstable fMRI signal intensity variations typical of the initial images. Pearson *r* coefficients per ROI pairs were subjected to a voxelwise *z*-transformation and exported for seed-based functional connectivity and network analyses in MATLAB (MathWorks). Composite functional connectivity maps were generated in AFNI for cortical and subcortical seed regions to determine the quality and consistency of resting-state correlation data across groups (see Fig. 3, representative group-level statistical map for rats in the chamber exposure control group).

### Network analyses

As an additional resting-state fMRI analysis method, we calculated basic graph theory metrics to assess the topology of functional connectivity networks. This approach has previously been applied to quantitatively determine salient features of the arrangement of functional correlations between brain regions in studies of cocaine, heroin, methamphetamine, and alcohol use disorders (Ahmadlou et al., 2013; Jiang et al., 2013; Wang et al., 2015; Sjoerds et al., 2017). Resting-state fMRI data were analyzed using Brain Connectivity Toolbox for MATLAB (Rubinov and Sporns, 2010). Symmetrical connectivity graphs with a total 11,175 matrix entries were first organized in MATLAB [graph size = *n*(*n* − 1)/2, where *n* is the number of nodes represented in the graph, or 150 ROIs]. The *z*-score values of the graphs were thresholder for each subject to create matrices with equal densities (e.g., *z* values in the top 15% of all possible correlation coefficients). Matrix *z* values were normalized by the highest *z* score, such that all matrices had edge weight values ranging from 0 to 1. Node strength (the sum of edge weights), clustering coefficient (the degree to which nodes cluster together in groups), the average shortest path length (the potential for communication between pairs of structures), modularity (the degree to which the network may be subdivided into clearly delineated groups or communities), and small worldness (the degree to which functional brain networks deviate from randomly connected networks) were calculated for weighted or unweighted graphs (Newman, 2003; Newman and Girvan, 2004; Boccaletti et al., 2006; Saramäki et al., 2007; Humphries and Gurney, 2008).

The small world (sw) index was determined by comparing rat functional connectivity networks to an average of 10 null hypothesis networks per rat (Watts and Strogatz, 1998). Thus, the ratio for clustering coefficients and path lengths of rat brain relative to null networks were calculated. The ratio of clustering coefficients is known as γ, which for a small world network is [mt]1 (Humphries and Gurney, 2008). The ratio of average path length is referred to as λ, which for a small world network is close to 1. The sw parameter is the ratio of γ/λ, with a sw >1, indicative of small world topology (typical of real-world networks), and sw ∼1, indicative of a random network (Erdös and Rényi, 1960). Brain networks were visualized using BrainNet (Xia et al., 2013). The 3D networks were generated with undirected edges weights (*E*_undir_) ≥0.3. In these brain networks (or rat brain connectomes), the node size and color were scaled by the node strength, and edges were scaled by *z* scores.

### Statistical analyses

A repeated-measures ANOVA was used to assess whether cocaine intake changed over the course of the 14 d of self-administration. To determine whether there was a difference in the number of nose pokes in the active versus inactive hole between self-administration groups across the course of self-administration, a three-factor repeated-measures ANOVA was used, with group (cocaine versus sucrose) as the between-subjects factor and nose poke (active versus inactive) and day as the within-subjects factors.

Global and brain region-specific network metrics and correlation coefficients were analyzed in MATLAB using a two-factor ANOVA with repeated measures (group × time). The study design included three imaging sessions [baseline, 1 d of Abs (1d Abs), and 14d of Abs (14d Abs)] and three groups (cocaine, sucrose, and chamber exposure). We first conducted statistical analyses of global measures of node strength, clustering coefficient, average path length, and small worldness. After this first analysis, statistical comparisons were made on ROI-specific measures of clustering coefficient. We focused on clustering coefficient because, in contrast to node strength, mean path length, and small worldness, the global clustering coefficient (for the entire brain) was significantly affected by cocaine SA (see Results). As indicated above, the clustering coefficient is a measurement of the tendency for any two neighbors of a node to be connected to each other (Rubinov and Sporns, 2010). For the ROI-specific analyses, 150 repeated-measures ANOVAs were conducted using custom scripts written in MATLAB, and the resulting *p* values were corrected for multiple comparisons using a false discovery rate (FDR) method (Storey, 2002). Similarly, *z*-transformed correlation coefficients were converted from symmetrical matrix format to vector form and a repeated-measures ANOVA was conducted per entry (11,175 pairwise correlation values), with final *p* values FDR corrected. A Tukey’s multiple-comparison test was used to assess the differences in means of all group/pairwise comparisons of node strength. (See Figs. 5, 6 and Tables 1 and 2, for results for *post hoc* analyses.) Significant session × group interactions are reported, and differences between the groups (cocaine, sucrose, and chamber exposure) are jointly dependent on group and imaging sessions. This statistical approach is consistent with the major objective of this study, which was to determine the effect of cocaine self-administration on network connectivity that varies as a function of time point (before or after self-administration).

**Table 1.** Regions showing significant bilateral group × session interactions for clustering coefficient

Regions	Baseline			1d Abs			14d Abs		
	Chamber control	Sucrose SA	Cocaine SA	Chamber control	Sucrose SA	Cocaine SA	Chamber control	Sucrose SA	Cocaine SA
CeA	0.27 ± 0.02	0.33 ± 0.06	0.35 ± 0.02	0.29 ± 0.03	0.47 ± 0.06	0.43 ± 0.04*	0.41 ± 0.05	0.31 ± 0.02	0.39 ± 0.06
BaA	0.28 ± 0.02	0.35 ± 0.04	0.35 ± 0.03	0.28 ± 0.03	0.41 ± 0.05	0.41 ± 0.05*	0.36 ± 0.04	0.30 ± 0.01	0.31 ± 0.04
NAc	0.25 ± 0.04	0.33 ± 0.06	0.30 ± 0.03	0.31 ± 0.05	0.42 ± 0.05	0.49 ± 0.08*	0.41 ± 0.06	0.28 ± 0.03	0.33 ± 0.03
dHPC	0.32 ± 0.04	0.42 ± 0.07	0.34 ± 0.04	0.32 ± 0.05	0.45 ± 0.11	0.46 ± 0.03*	0.41 ± 0.03	0.38 ± 0.04	0.31 ± 0.02
VP	0.28 ± 0.02	0.34 ± 0.05	0.36 ± 0.05	0.30 ± 0.06	0.43 ± 0.04	0.50 ± 0.08*	0.42 ± 0.04	0.31 ± 0.02	0.35 ± 0.04
GP	0.28 ± 0.03	0.31 ± 0.04	0.37 ± 0.04	0.34 ± 0.05	0.43 ± 0.01	0.47 ± 0.07	0.39 ± 0.05	0.27 ± 0.03	0.30 ± 0.05
LH	0.28 ± 0.02	0.34 ± 0.03	0.40 ± 0.03	0.30 ± 0.02	0.39 ± 0.05	0.45 ± 0.05*	0.35 ± 0.04	0.31 ± 0.03	0.32 ± 0.04
PV Thal	0.31 ± 0.02	0.41 ± 0.05	0.39 ± 0.05	0.34 ± 0.05	0.51 ± 0.04	0.55 ± 0.07*	0.42 ± 0.04	0.30 ± 0.02	0.34 ± 0.05
AP Thal	0.31 ± 0.01	0.45 ± 0.06	0.40 ± 0.04	0.32 ± 0.04	0.46 ± 0.08	0.58 ± 0.06*	0.40 ± 0.03	0.30 ± 0.04	0.33 ± 0.03
RN Thal	0.29 ± 0.04	0.33 ± 0.07	0.39 ± 0.04	0.32 ± 0.05	0.43 ± 0.05	0.41 ± 0.04	0.45 ± 0.06	0.28 ± 0.04	0.34 ± 0.05
MD Thal	0.35 ± 0.03	0.42 ± 0.04	0.33 ± 0.03	0.32 ± 0.05	0.41 ± 0.05	0.48 ± 0.06*	0.36 ± 0.03	0.32 ± 0.02	0.30 ± 0.02
VL Thal	0.32 ± 0.02	0.35 ± 0.03	0.38 ± 0.03	0.32 ± 0.04	0.45 ± 0.04*	0.47 ± 0.05*	0.40 ± 0.03	0.30 ± 0.00	0.38 ± 0.01
VPM Thal	0.27 ± 0.04	0.35 ± 0.06	0.35 ± 0.03	0.32 ± 0.03	0.57 ± 0.06*	0.39 ± 0.08*	0.38 ± 0.05	0.32 ± 0.03	0.34 ± 0.04
LP Thal	0.33 ± 0.02	0.41 ± 0.08	0.35 ± 0.04	0.31 ± 0.03	0.47 ± 0.10*	0.54 ± 0.04*	0.37 ± 0.03	0.26 ± 0.04	0.31 ± 0.02
Pf Thal	0.30 ± 0.03	0.39 ± 0.05	0.31 ± 0.03	0.29 ± 0.04	0.46 ± 0.01*	0.51 ± 0.06*	0.42 ± 0.07	0.32 ± 0.01	0.34 ± 0.02
LG Thal	0.33 ± 0.03	0.41 ± 0.04	0.33 ± 0.05	0.33 ± 0.05	0.47 ± 0.04	0.52 ± 0.04*	0.40 ± 0.06	0.33 ± 0.03	0.37 ± 0.04
PrL	0.33 ± 0.02	0.36 ± 0.05	0.30 ± 0.02	0.31 ± 0.05	0.41 ± 0.02	0.40 ± 0.03	0.43 ± 0.06	0.31 ± 0.02	0.36 ± 0.04
IL	0.30 ± 0.03	0.28 ± 0.02	0.30 ± 0.01	0.31 ± 0.06	0.49 ± 0.12	0.43 ± 0.06	0.43 ± 0.08	0.28 ± 0.03	0.32 ± 0.04
M2	0.29 ± 0.03	0.28 ± 0.00	0.39 ± 0.05	0.29 ± 0.05	0.33 ± 0.07	0.46 ± 0.06	0.50 ± 0.08	0.31 ± 0.03	0.36 ± 0.03
S1 jw	0.29 ± 0.03	0.39 ± 0.04	0.28 ± 0.02	0.31 ± 0.04	0.42 ± 0.04	0.38 ± 0.08	0.43 ± 0.04	0.30 ± 0.03	0.34 ± 0.05
S1 ul	0.28 ± 0.02	0.31 ± 0.01	0.37 ± 0.05	0.29 ± 0.03	0.38 ± 0.05	0.44 ± 0.07*	0.40 ± 0.03	0.30 ± 0.04	0.35 ± 0.04
S1 Sh	0.29 ± 0.02	0.36 ± 0.03	0.33 ± 0.03	0.33 ± 0.05	0.44 ± 0.05	0.49 ± 0.07*	0.38 ± 0.04	0.29 ± 0.02	0.32 ± 0.04
S2	0.28 ± 0.03	0.33 ± 0.02	0.39 ± 0.05	0.31 ± 0.05	0.39 ± 0.06	0.48 ± 0.05*	0.40 ± 0.03	0.26 ± 0.04	0.33 ± 0.06

Data are shown as the mean ± SEM for sucrose or cocaine SA rats and chamber exposure controls at baseline, 1d Abs, and 14d Abs. CeA, Central amygdala; BaA, basal amygdala; dHPC, dorsal hippocampus; VP, ventral pallidum; GP, globus pallidus; LH, lateral hypothalamus; Thal, thalamus; PV, paraventricular; AP, anteroposterior; RN, reticular nucleus; MD, mediodorsal; VL, ventrolateral; VPM, ventroposteromedial; LP, lateroposterior; Pf, parafascicular; LG, lateral genticulate; PrL, prelimbic; IL, infralimbic; M2, secondary motor; S1, primary somatosensory cortex; jw, jaw region, ul, upper lip; Sh, shoulder; S2, secondary somatosensory cortex. All regions in the table showed significant group × session interactions with a repeated-measures two-factor ANOVA (α < 0.05).

Asterisks represent the results of Tukey’s multiple-comparison *post hoc* test: *difference from chamber exposure controls; **difference between cocaine and sucrose groups.

**Table 2.** Regions showing significant lateralized group × session interactions for clustering coefficient

Regions	Baseline			1d Abs			14d Abs		
	Chamber control	Sucrose SA	Cocaine SA	Chamber control	Sucrose SA	Cocaine SA	Chamber control	Sucrose SA	Cocaine SA
LaA (R)	0.29 ± 0.03	0.32 ± 0.04	0.30 ± 0.04	0.30 ± 0.02	0.40 ± 0.06	0.47 ± 0.06*	0.40 ± 0.05	0.33 ± 0.02	0.34 ± 0.03
MeA (R)	0.31 ± 0.03	0.38 ± 0.06	0.41 ± 0.02	0.32 ± 0.03	0.43 ± 0.04	0.49 ± 0.06*	0.41 ± 0.06	0.27 ± 0.02	0.32 ± 0.04
DMS (L)	0.30 ± 0.04	0.29 ± 0.05	0.39 ± 0.05	0.31 ± 0.05	0.46 ± 0.04	0.49 ± 0.05*	0.44 ± 0.04	0.28 ± 0.02	0.37 ± 0.04
LSept (R)	0.30 ± 0.02	0.29 ± 0.03	0.39 ± 0.04	0.35 ± 0.08	0.37 ± 0.09	0.56 ± 0.07*	0.42 ± 0.04	0.26 ± 0.01	0.32 ± 0.04
AHA (R)	0.28 ± 0.03	0.35 ± 0.03	0.34 ± 0.03	0.30 ± 0.03	0.49 ± 0.04*	0.51 ± 0.07*	0.41 ± 0.06	0.34 ± 0.05	0.36 ± 0.05
MB (R)	0.28 ± 0.03	0.37 ± 0.05	0.32 ± 0.02	0.31 ± 0.03	0.41 ± 0.09	0.51 ± 0.09*	0.42 ± 0.05	0.32 ± 0.02	0.36 ± 0.04
VM Thal (R)	0.34 ± 0.03	0.43 ± 0.04	0.40 ± 0.03	0.31 ± 0.03	0.51 ± 0.05*	0.52 ± 0.07*	0.40 ± 0.06	0.28 ± 0.04	0.36 ± 0.03
PThal (R)	0.34 ± 0.03	0.36 ± 0.07	0.38 ± 0.03	0.32 ± 0.03	0.45 ± 0.08	0.47 ± 0.04*	0.35 ± 0.03	0.29 ± 0.02	0.33 ± 0.03
MG Thal (L)	0.31 ± 0.02	0.38 ± 0.04	0.41 ± 0.04	0.36 ± 0.07	0.45 ± 0.05	0.52 ± 0.05	0.45 ± 0.09	0.38 ± 0.04	0.35 ± 0.04
cRSC (L)	0.39 ± 0.04	0.34 ± 0.03	0.35 ± 0.05	0.38 ± 0.05	0.60 ± 0.07*	0.51 ± 0.05	0.46 ± 0.04	0.36 ± 0.05	0.36 ± 0.02
M1 (L)	0.26 ± 0.02	0.29 ± 0.02	0.29 ± 0.03	0.31 ± 0.04	0.40 ± 0.07	0.38 ± 0.05	0.45 ± 0.05	0.32 ± 0.02	0.33 ± 0.04
S1 Tr (R)	0.31 ± 0.03	0.44 ± 0.09	0.40 ± 0.06	0.26 ± 0.04	0.43 ± 0.07	0.48 ± 0.06*	0.48 ± 0.09	0.38 ± 0.03	0.32 ± 0.02
PRhC (L)	0.30 ± 0.02	0.27 ± 0.04	0.30 ± 0.03	0.34 ± 0.02	0.60 ± 0.10*	0.41 ± 0.04**	0.39 ± 0.05	0.28 ± 0.02	0.36 ± 0.04
MRN (L)	0.35 ± 0.03	0.35 ± 0.05	0.38 ± 0.04	0.37 ± 0.04	0.44 ± 0.03	0.53 ± 0.04*	0.38 ± 0.03	0.43 ± 0.03	0.34 ± 0.03
Cer2 (L)	0.38 ± 0.04	0.39 ± 0.04	0.33 ± 0.02	0.33 ± 0.05	0.49 ± 0.02*	0.46 ± 0.05*	0.51 ± 0.05	0.32 ± 0.02*	0.35 ± 0.03*

Data are shown as the mean ± SEM for sucrose or cocaine SA rats and chamber exposure controls at baseline, 1d Abs, and 14d Abs. R, Right; L, left; LaA, lateral amygdala; MeA, medial amygdala; DMS, dorsomedial striatum; LSept, lateral septum; AHA, anterior hypothalamic area; MB, mammillary bodies; Thal, thalamus; VM, ventromedial; Pthal, posterior thalamus; MG, medial genticulate; cRSC, caudal retrosplenial cortex; M1, primary motor cortex; S1, primary somatosensory cortex; Tr, trunk region; PRhC, perirhinal cortex; MRN, midbrain reticular nucleus; Cer2, 2^nd^ cerebellar lobule. All regions in table showed significant group x session interactions with a repeated-measures two factor ANOVA (α < 0.05).

Asterisks represent results of Tukey’s multiple-comparison *post hoc* test: *difference from chamber exposure controls; **difference between cocaine and sucrose groups..

## Results

### Escalation of cocaine self-administration

**Figure 2.**
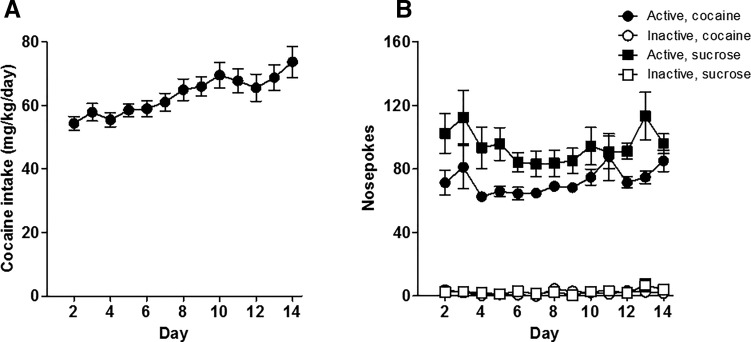
Self-administration behavior. ***A***, Rats increased their cocaine intake across 14 d of self-administration with daily 6 h access. ***B***, Both the cocaine and sucrose self-administration groups preferred the active nose poke over the inactive nose poke. Data are presented as the mean ± SEM.

A repeated-measures ANOVA showed that rats in the cocaine group escalated their cocaine intake over the 14 d of self-administration (Fig. 2*A*; main effect of day, *F*_(13,91)_ = 3.67, *p* < 0.001). In addition, a three-factor repeated-measures ANOVA (nose poke × group × day) showed that the cocaine and sucrose groups chose the active over the inactive nose poke (Fig. 2*B*; main effect of nose poke, *F*_(1,11)_ = 585.8, *p* < 0.001) to a comparable degree across the 14 d of self-administration (nose poke × group, *F*_(1,11)_ = 9.4, *p* < 0.05; nose poke × day × group, *F*_(13,143)_ = 0.42, *p* = 0.96). Note that escalation in the sucrose group could not be analyzed because the sucrose intake of each rat depended on the daily cocaine intake in their matched cocaine counterparts. As described above, this experimental design allows comparisons of functional connectivity resulting from cocaine versus sucrose intake without the confound of differing amounts of self-administration experience.

### Resting-state functional connectivity is altered by extended access to cocaine self-administration in rats

**Figure 3.**
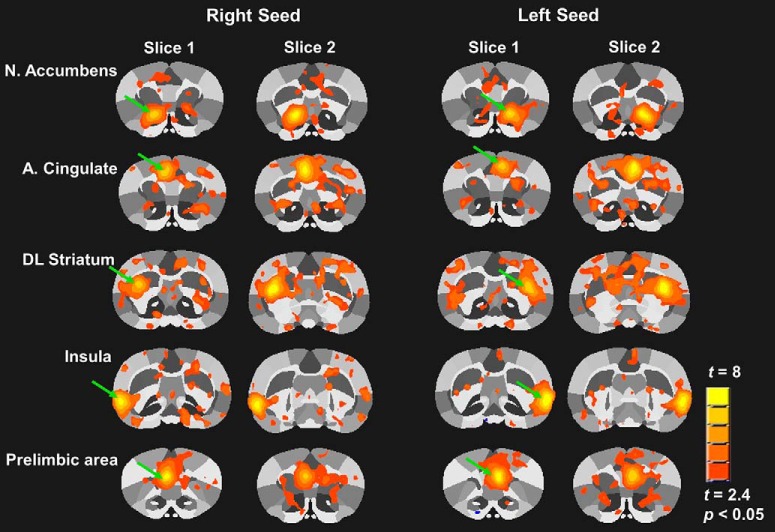
Seed-based functional connectivity maps illustrating left and right hemisphere seeds for striatal and cortical regions. Maps are composite statistical maps from the chamber exposure control group at baseline. Regions showing significant functional connectivity with the seed region are displayed (uncorrected threshold, *t* > 2.4, *p* < 0.05). Green arrows indicate the seed region in each map. Each region shows 2 contiguous slices of 12 total coronal slices scanned from rostral to caudal in the brain.

**Figure 4.**
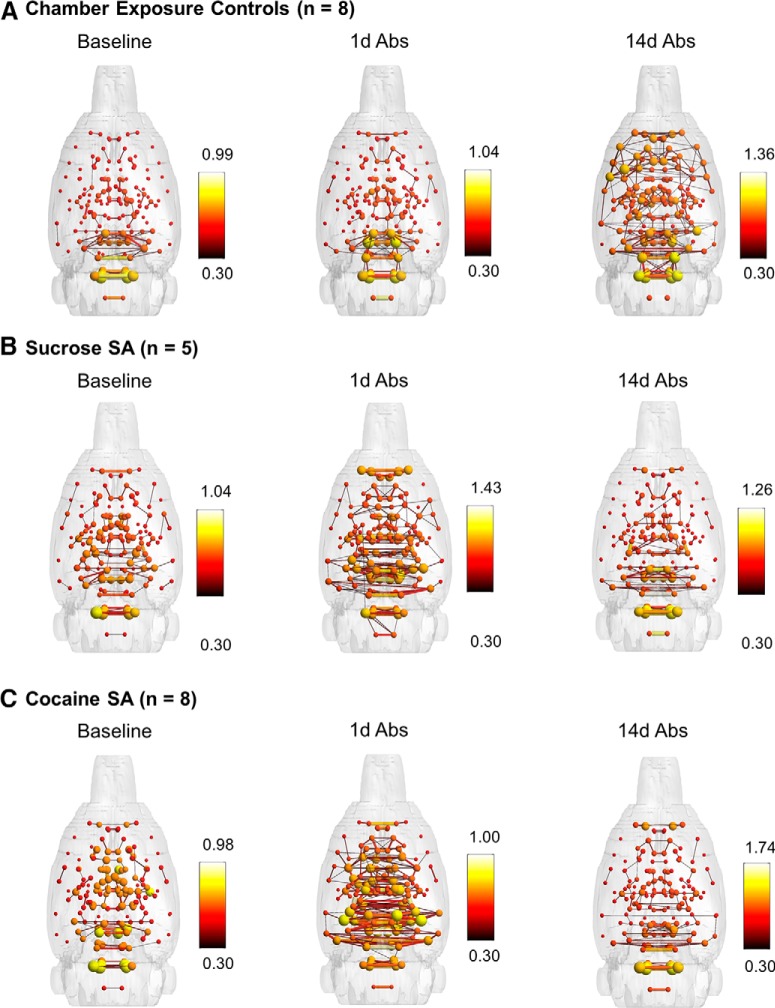
Three-dimensional functional connectivity maps of the rat brain illustrating significant effects of sucrose or cocaine self-administration and Abs. ***A***, Chamber exposure controls showed no change at 1d Abs and an increase in cortical functional connectivity at 14d Abs. ***B***, The sucrose group largely showed increased connectivity in subcortical areas at 1d Abs. ***C***, The cocaine group showed increased functional connectivity in subcortical areas, including thalamic, hypothalamic, and forebrain regions at 1d Abs, whereas decreases were observed at 14d Abs. All maps are composites of n = 5-8 rats and are set at a correlation threshold (edge weight) value of 0.3. Spheres represent node strength, and line thicknesses represent edge weights.

Group-level statistical maps of functional connectivity between mesocorticolimbic areas of chamber exposure control rats are shown in Figure 3. These highlight the functional connectivity patterns observed in the baseline presurgical imaging session (i.e., in naïve rats that go on to be chamber exposure controls). Figure 4*A–C* shows 3D rat brain shells with representations of node strength (spheres) and edge weights (lines connecting spheres) for the chamber exposure control, sucrose and cocaine groups. Connectivity patterns are shown for each group at baseline (before surgery and self-administration) and after 1d Abs and 14d Abs. Although these are qualitative, the maps suggest that chamber exposure control rats showed consistent functional connectivity patterns between sessions at baseline and 1d Abs, but increased connectivity at 14d Abs. In contrast, sucrose and cocaine groups had increased connectivity relative to baseline at 1d Abs, but connectivity was reduced again by 14d Abs.

### Arrangement of nodal interactions is affected by cocaine self-administration, as demonstrated by increased clustering, small worldness and modularity over the course of abstinence

**Figure 5.**
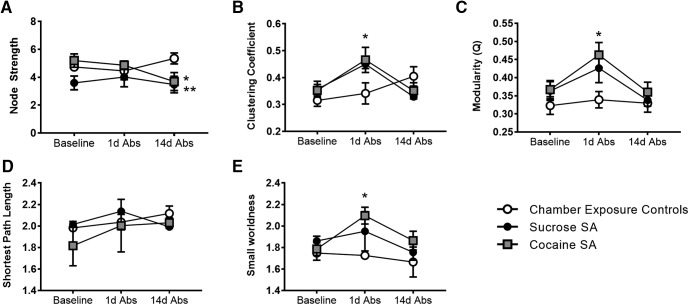
Cocaine self-administration increases modularity, clustering, and small worldness at 1d Abs. ***A***, Node strength. ***B***, Clustering coefficient. ***C***, Modularity. ***D***, Average path length. ***E***, Small worldness. All data are the mean ± SE. *Cocaine group vs control; **sucrose group vs control at 14 Abs. Two-way repeated-measures ANOVA used with Tukey’s multiple-comparison post hoc test (α < 0.05).

Global network metrics were analyzed, and the results are shown in Figure 5*A–E*. Although the mean path length and the small world index did not show a significant session × group interaction, node strength showed a trend toward a session × group interaction (*F*_(4,36)_ = 2.2, *p* = 0.08; a Tukey’s *post hoc* test indicated reduced node strength in cocaine and sucrose rats relative to chamber exposure controls, *p* < 0.05; Fig. 5*A*). Analysis of the clustering coefficient showed a significant session × group interaction (*F*_(4,36)_ = 2.9, *p* = 0.03; Fig. 5*B*). The Tukey’s multiple-comparison test indicated that the clustering coefficient was higher in cocaine SA rats than chamber exposure controls on 1d Abs (*p* = 0.01). Sucrose showed a similar, albeit nonsignificant, trend (*p* = 0.08) relative to chamber exposure controls. We should note that while the session × group interaction was not significant for the small world index, there was a significant main effect of group (*F*_(2,18)_ = 3.9, *p* = 0.03). The Tukey’s *post hoc* comparison test revealed an effect-like clustering coefficient such that cocaine SA rats had a significantly greater small world index than chamber exposure controls at 1d Abs (*p* = 0.009; Fig. 5*E*). Given that at 1d Abs there was an increase in clustering in cocaine SA compared with chamber exposure controls, we next determined whether this was also associated with a rearrangement of nodes into highly interconnected subsets by calculating the modularity index Q (Newman and Girvan, 2004). In a modular organization, subsets of nodes show high interconnectivity relative to chance, forming communities (Rubinov and Sporns, 2010). We observed that cocaine SA rats on 1d Abs had a greater Q value relative to their own baseline (main effect session: *F*_(2,36)_ =4.5, *p* = 0.02) and to chamber exposure controls at 1d Abs (main effect group: *F*_(2,36)_ = 6.0, *p* = 0.01; Fig. 5*C*; no significant group × session interaction). Sucrose rats showed a similar nonsignificant trend (*p* = 0.10; Fig. 5*C*). Together with the 3D connectomic maps in Figure 4, changes in these global topological measures indicate that chronic cocaine SA reorganizes functional connectivity patterns in the rat brain.

### Cocaine and sucrose self-administration increase clustering coefficient in cortical, striatal, amygdala, thalamic, and hypothalamic areas of the rat brain

**Figure 6.**
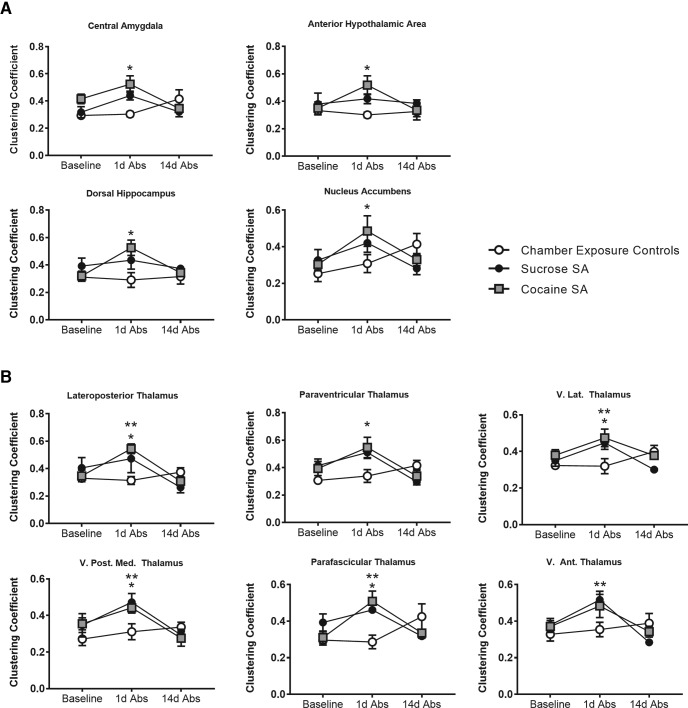
Cocaine or sucrose self-administration increased clustering across limbic, cortical, and thalamic regions at 1d Abs. *Cocaine group vs chamber exposure controls at 1d Abs; **sucrose group vs chamber exposure controls at 1d Abs. Repeated-measures ANOVA with Tukey’s multiple-comparison test (α < 0.05).

In addition to the global functional network analyses described above, we further assessed the clustering coefficient for individual ROIs. The clustering coefficient was analyzed for 150 ROIs, divided equally between left and right hemisphere representations. Following multiple-comparisons (FDR) correction, 23 ROIs showed significant bilateral session × group interactions (both left and right representations had the same significant outcome; Table 1). These ROIs included limbic areas, basal forebrain, and cortical and thalamic regions, some of which are shown in Figure 6. There was a significant session × group interaction in the central (*F*_(4,36)_ = 2.8, *p* = 0.04) and basal amygdala (*F*_(4,36)_ = 2.7, *p* = 0.04); nucleus accumbens (*F*_(4,36)_ = 2.1, *p* = 0.04); dorsal hippocampus (*F*_(4,36)_ = 2.0, *p* = 0.04); ventral pallidum (*F*_(4,36)_ = 2.5, *p* = 0.04); globus pallidus (*F*_(4,36)_ = 2.0, *p* = 0.04); lateral hypothalamus (*F*_(4,36)_ = 2.4, *p* = 0.04); paraventricular (*F*_(4,36)_ = 2.8, *p* = 0.04), anteroposterior (*F*_(4,36)_ = 4.9, *p* = 0.01), reticular (*F*_(4,36)_ = 2.5, *p* = 0.04), mediodorsal (*F*_(4,36)_ = 3.3, *p* = 0.03), ventrolateral (*F*_(4,36)_ = 3.4, *p* = 0.03), ventroposteromedial (*F*_(4,36)_ = 2.6, *p* = 0.04), lateroposterior (*F*_(4,36)_ = 2.9, *p* = 0.04), parafascicular (*F*_(4,36)_ = 3.3, *p* = 0.03), and lateral genticulate thalamic nuclei (*F*_(4,36)_ = 2.8, *p* = 0.04); the prelimbic (PL; *F*_(4,36)_ = 2.1, *p* = 0.04), infralimbic (*F*_(4,36)_ = 2.7, *p* = 0.03), and secondary motor cortices (*F*_(4,36)_ = 2.7, *p* = 0.04); jaw (*F*_(4,36)_ = 2.4, *p* = 0.04), upper lip (*F*_(4,36)_ = 2.0, *p* = 0.04), and shoulder primary somatosensory cortices (*F*_(4,36)_ = 2.3, *p* = 0.04); and secondary somatosensory cortex (*F*_(4,36)_ = 2.2, *p* = 0.04).

An additional 15 ROIs showed significant unilateral session × group interactions (only one hemispheric representation had a significant outcome; Table 2). These included ROIs associated anatomically with the above regions, such as right lateral (*F*_(4,36)_ = 2.4, *p* = 0.04) and right medial amygdala (*F*_(4,36)_ = 4.6, *p* = 0.02); left dorsomedial striatum (*F*_(4,36)_ = 2.4, *p* = 0.04); right lateral septum (*F*_(4,36)_ = 2.2, *p* = 0.04); right anterior hypothalamic area (*F*_(4,36)_ = 2.2, *p* = 0.04); right mammillary bodies (*F*_(4,36)_ = 2.2, *p* = 0.04); right ventromedial (*F*_(4,36)_ = 3.9, *p* = 0.03), right posterior (*F*_(4,36)_ = 2.2, *p* = 0.04), and left medial genticulate nuclei of the thalamus (*F*_(4,36)_ = 2.6, *p* = 0.04); left caudal retrosplenial (*F*_(4,36)_ = 3.9, *p* = 0.04) and left primary motor cortices (*F*_(4,36)_ = 2.6, *p* = 0.04); right trunk area of somatosensory cortex (*F*_(4,36)_ = 2.8, *p* = 0.04); left perirhinal cortex (*F*_(4,36)_ = 5.2, *p* = 0.04); left midbrain reticular nucleus (*F*_(4,36)_ = 2.3, *p* = 0.04); and left second lobule of the cerebellum (*F*_(4,36)_ = 5.6, *p* = 0.02).

**Figure 7.**
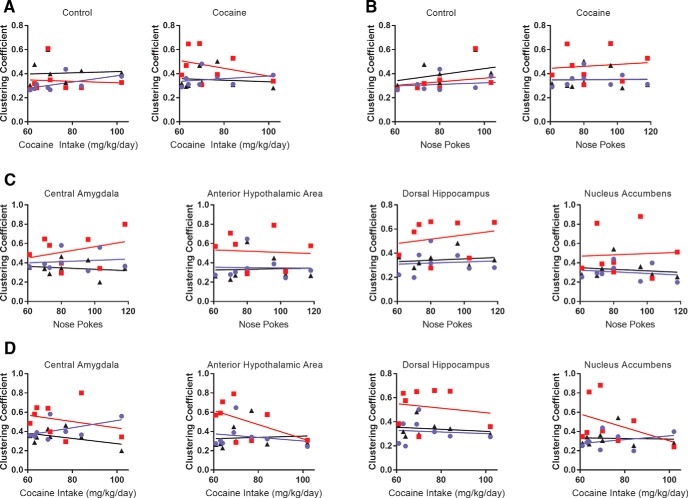
No correlation between cocaine self-administration and global and regional brain clustering coefficient was observed. ***A***, Cocaine intake data for global clustering coefficient in chamber exposure controls and cocaine-treated rats. ***B***, Nose-poke data for the same groups as in ***A***. ***C***, ***D***, Nose-poke and cocaine intake correlations with regional clustering coefficients of cocaine rats. Blue circles, Baseline day; red squares, 1d Abs; black triangles, 14d Abs.

Across these regions, Tukey’s *post hoc* multiple comparison revealed an increase in clustering coefficient in cocaine SA rats relative to chamber exposure controls at 1d Abs (Tables 1, 2). In many of the same ROIs, particularly in thalamic nuclei, sucrose had a similar effect at 1d Abs. Examples of regions showing this pattern are shown in Figure 6*B*. The central amygdala, the hypothalamus, dorsal hippocampus, nucleus accumbens, and the thalamus of cocaine SA rats had a significantly greater clustering coefficient than chamber exposure controls at 1 Abs (cocaine SA vs chamber exposure controls on 1d Abs, *p* < 0.05; Fig. 6*A*). Similar effects were observed with sucrose SA, but this was mostly significant only for thalamic nuclei (Fig. 6*B*, Tables 1, 2). Finally, for the ROIs showing significant effects of cocaine SA at 1d Abs, we determined whether there was a stepwise linear correlation between the cocaine SA and global and ROI-specific clustering coefficient. This linear regression analysis used behavioral data for total cocaine intake and the number of lever presses for each rat on the final day of the 14 d cocaine SA session and compared it with imaging results on the following day (1d Abs). Results are summarized in Figure 7. Although slope elevations were significantly different in cocaine SA rats when comparing baseline with 1d Abs from cocaine, no significant correlations with behavior were observed. We compared other global network metrics, and no correlations with behavior were observed.

### Strength of functional interactions is not significantly affected by cocaine or sucrose SA

Node strength was further analyzed as an indicator of the strength of all significant correlations for each ROI. This metric was calculated for each of the 150 ROIs as the sum of all its edge weights (correlation coefficients). In several regions, we observed an effect of cocaine or sucrose SA similar to that seen with the global node strength metric (Fig. 5*A*) such that there was reduced node strength at 14d Abs. However, following FDR correction, there were no significant effects of cocaine or sucrose SA on any of the 150 ROIs in any of the imaging sessions. Similarly, none of the standard seed-based functional connectivity measurements (correlation coefficients) revealed significant effects of cocaine or sucrose following FDR correction.

To summarize, 2 weeks of extended access cocaine SA resulted in an increased clustering coefficient and modularity at 1d Abs, but not 14d Abs. Sucrose had a similar effect on these network metrics, albeit to a less robust degree than cocaine. Overall, the results suggest that cocaine SA affected network metrics that reflect the arrangement of nodal interactions but had less of an impact on functional connectivity measurements, reflecting the strength of connectivity between ROIs.

## Discussion

The present study adds to data supporting the use of resting-state functional connectivity as an *in vivo* biomarker for functional alterations related to cocaine use. In addition, the results emphasize the importance of including a predrug exposure baseline when analyzing neuroimaging-based neuroadaptations in the context of chronic substance use. With this study design, we observed significant increases in the clustering coefficient after 1 d of abstinence from cocaine self-administration. The clustering coefficient quantifies the number of connections neighboring a node, normalized to the total number of possible connections. This measure of connectivity is often interpreted as reflecting specialization of a node (or brain region), which in the present case could be due to changes in the pattern or arrangement of functional connectivity across the totality of ROIs analyzed in rats with a recent history of cocaine self-administration. To confirm that chronic cocaine exerted an impact on the arrangement of nodal interactions, we analyzed the modularity index and found that by 1d Abs, there was an increase in modular organization of functional connectivity with cocaine or sucrose SA. Such increases in clustering and modularity could be a result of a differential modification of network-level activity during early and late abstinence from cocaine self-administration (Volkow et al., 2013). Further, our results suggest that discrete nodes in amygdala, nucleus accumbens, thalamic subnuclei, and limbic and sensorimotor cortices may be central to this highly clustered, modular organization enhanced by cocaine or sucrose SA.

A surprising outcome of the present study was that there were no significant effects of cocaine or sucrose SA on the strength of functional connectivity after correcting for multiple comparisons. Standard functional connectivity methods use the correlation coefficient to compare the strength of BOLD signal interactions between pairs of a priori-selected brain areas. In many neuroimaging studies of cocaine use, withdrawal-dependent reductions in connectivity strength are observed in cocaine-dependent subjects relative to healthy control subjects (Gu et al., 2010). However, most studies assessing the effects of drugs of abuse on the resting-state signal are cross sectional and do not provide a baseline measurement to support the observed changes following drug use. In the present study design, we included measurements of baseline functional connectivity, and by doing so we observed a lack of effect of cocaine or sucrose SA on the strength of connectivity. In addition, a related network measure, node strength, was found not to vary significantly across sessions and between groups. Thus, the strength of connectivity (either using correlation coefficients or node strength) seems to be a less sensitive functional measure for the effects of cocaine or sucrose SA when considering initial baseline conditions. Instead, our data indicate that the arrangement of functional connectivity is affected by cocaine or sucrose self-administration, and this was indexed by increases in clustering and modularity. Rearrangement of neural circuits driving specific affective behaviors, following a temporal window of exposure, has been previously reported in other areas (Do-Monte et al., 2015), and this could be a mechanism of importance that merits investigation in the substance use field. The increased clustering and modular organization is observed in cocaine SA rats at a short-term abstinence interval, which is consistent with previous results showing increased small worldness in recently abstinent cocaine users (Wang et al., 2015). It is intriguing that most studies centered on long-term abstinence or withdrawal report reduced functional connectivity in cocaine users versus healthy controls (Hu et al., 2015). Therefore, there is a neurobiological rationale for such longer-term reductions, and the present findings may represent shorter-term adaptations that give way to reduced connectivity at longer (e.g., 30 d) abstinence durations (which were not measured here).

One notable region within the mesocorticolimbic circuit that was subject to the effects of cocaine on the organization of functional connectivity is the ventral striatum, which has been implicated in processing information about both natural and drug reinforcers (Volkow and Morales, 2015). Previous work has shown reduced gray matter in the striatum of chronic cocaine users (Barrós-Loscertales et al., 2011; Mackey and Paulus, 2013), and lower levels of glucose metabolism in the ventral striatum of rodents and nonhuman primates after cocaine self-administration (Porrino et al., 2002; Macey et al., 2004; Beveridge et al., 2006; Calipari et al., 2013). Further, neuroimaging studies in rodents have revealed a reduction in activity in the ventral striatum after chronic cocaine exposure (Febo et al., 2005; Gozzi et al., 2011). In line with this reduction, electrophysiological experiments have shown both *in vivo* and *in vitro* that chronic cocaine use causes a reduction in firing in the NAc during abstinence from cocaine SA (Thomas et al., 2001; Kourrich and Thomas, 2009; Mu et al., 2010; Cameron and Carelli, 2012; Saddoris et al., 2016). This reduction in activity may reflect decreases in the release of monoamine neurotransmitters, such as serotonin and DA. Indeed, amphetamine-induced increases in cerebral blood volume, an indirect proxy of resting-state brain function, are blunted in the ventral striatum of rats with a history of cocaine self-administration (Gozzi et al., 2011). Further, using fast-scan cyclic voltammetry, others have shown that cue-induced DA release in the NAc of rats is substantially altered after 30 d of abstinence from cocaine self-administration (Saddoris et al., 2016). Notably, in this study, as well as in others using *in vivo* electrophysiology, these alterations in ventral striatal activity and DA release within this region are linked to behavioral deficits such as impaired learning and goal-directed behavior (Cameron and Carelli, 2012; Saddoris and Carelli, 2014; Saddoris et al., 2016), suggesting that reduced ventral striatal activity may underlie maladaptive behavior observed in chronic cocaine users.

Another region affected by chronic cocaine SA that has been implicated in reward-related behavior is the amygdala. Prior neuroimaging studies have demonstrated that amygdala volume in cocaine users is significantly smaller than that of matched control subjects (Makris et al., 2004) and that reductions in amygdala cerebral blood flow are correlated with greater cocaine dependence (Wang et al., 2017). Consistent with these findings in humans, cocaine self-administration causes a decrease in amygdala glucose metabolism in both rats and nonhuman primates (Macey et al., 2004; Beveridge et al., 2006; Calipari et al., 2013). Importantly, amygdala integrity is critical for guiding adaptive decision-making behavior. For instance, lesions of the amygdala cause an increase in the choice of risky and disadvantageous options in rodent decision-making tasks (Zeeb and Winstanley, 2011; Orsini et al., 2015). Hence, cocaine-induced reductions in amygdala function may mediate decision-making deficits that are characteristic of chronic cocaine users (Aron and Paulus, 2007; Noël et al., 2013; Worhunsky et al., 2017). In support of this contention, *in vivo* electrophysiology experiments in rodents show that cocaine exposure alters the encoding properties of amygdala neurons. For example, chronic cocaine prevents neural activity in the amygdala from tracking representations of behavioral outcomes (Stalnaker et al., 2007a,b; Stalnaker et al., 2009) and attenuates anticipatory amygdala activity during delays preceding reward delivery (Zuo et al., 2012). Although the mechanism by which cocaine causes these reductions in amygdala function is not clear, recent work shows that cocaine self-administration in rodents causes an increase in basal GABAergic transmission in the central nucleus of the amygdala (Kallupi et al., 2013). Notably, this increase in GABAergic transmission in the central amygdala was observed immediately after the cessation of cocaine self-administration (after 1 d of abstinence); future studies are therefore warranted to determine whether augmented baseline GABA transmission persists further into abstinence.

In addition to the ventral striatum and amygdala, functional connectivity in several thalamic nuclei was altered relative to baseline. Furthermore, there were changes in functional connectivity within thalamic nuclei as well as with other regions involved in affective processing (e.g., amygdala) in both cocaine and sucrose groups relative to chamber exposure controls. The cocaine-induced functional changes in the thalamus are consistent with those shown in previous neuroimaging work showing hypoactivation of the thalamus in cocaine users (Tomasi et al., 2007, 2010), an effect that has been attributed to decreased dopaminergic activity within this region (Volkow et al., 1997; Tomasi et al., 2007). Animal studies have largely confirmed this finding using measures of glucose metabolism and basal cerebral blood volume. For example, there is a decrease in glucose metabolism in the mediodorsal, anterior, and intralaminar thalamic nuclei in nonhuman primates with a history of cocaine self-administration (Beveridge et al., 2006). Similarly, Gozzi et al. (2011) observed decreased cerebral blood volume (another surrogate marker of neural activity) in the reticular thalamic nuclei in rats after cocaine self-administration. Notably, there have also been reports of increased glucose metabolism in several thalamic nuclei after cocaine self-administration in nonhuman primates (Porrino et al., 2002; Macey et al., 2004); this discrepancy, however, may be due to differences in experimental design, including the imaging time point, the self-administration regimen, and the species studied.

Irrespective of these differences, these data, in conjunction with those in the current study, clearly demonstrate that chronic cocaine use perturbs thalamic activity. Under normal conditions, the thalamus is considered to act as a sensory gateway and is necessary for the allocation of attention via its connectivity with and across cortical structures (Fan et al., 2005; Sherman, 2005; Nakajima and Halassa, 2017). Thus, cocaine-induced thalamic alterations may underlie the attentional deficits and impairments in sensorimotor processing seen in cocaine users. Consistent with this, Tomasi et al. (2007) reported reduced thalamic activation in cocaine users performing a sustained visuospatial attention task. Collectively, these data highlight the need to further investigate the role of the thalamus in the context of cocaine use, as dysregulation within this structure may explain some of the cognitive and behavioral deficits commonly observed in cocaine users.

Human neuroimaging studies are limited in their ability to determine whether changes in functional connectivity are due specifically to substance use or instead whether they reflect pre-existing vulnerabilities that predispose some individuals to substance use. The use of animal models circumvents this problem and allows for controlled measurements of functional connectivity at discrete time points before and after drug exposure. To date, only one other study (Lu et al., 2014) has used a rodent model of cocaine self-administration and assessed changes in resting-state functional connectivity. After 1 month of abstinence, there was a decrease in connectivity between the PL area of the medial PFC and the right endopeduncular nucleus (EPN), and between the NAc core and the dorsomedial PFC (dmPFC) in rats that previously self-administered cocaine, compared with both a sucrose group and home-cage controls. In the current study, there were no changes between the PL and EPN at any time point after cocaine self-administration, nor were there changes in connectivity between other areas of the cortex and striatum. The discrepancy between these findings may be due to procedural differences in self-administration. For instance, in the current study, rats underwent self-administration for 14 d under long-access conditions (6 h) at a dose of 1.0 mg/kg. In the study by Lu et al. (2014), self-administration consisted of both short-access (1 h) and long-access conditions across a total of 24 d at a dose of 0.75 mg/kg. Another notable difference is that changes in functional connectivity in abstinence were assessed only at 1 and 14 d after self-administration in the current study, whereas Lu et al. (2014) imaged rats at 30 d of abstinence. Thus, it is possible that if the rats in the current study were imaged at a later time point, reductions between the PL and EPN and between the dmPFC and NAc core may have been observed. Finally, it is important to note that in contrast to the current study, Lu et al. (2014) did not collect measures of functional connectivity before self-administration (i.e., at baseline) or immediately following self-administration. Thus, the differences in connectivity between the PL and EPN and between the dmPFC and NAc core between the present study and that of Lu et al. (2014) may reflect a pre-existing neural phenotype rather than a long-term consequence of cocaine self-administration. In line with this speculation, Lu et al. (2014) also reported that there was a positive correlation in connectivity strength between dmPFC and NAc core and the escalation of intake: relative to all control rats, the reduction in strength in this circuit was greatest in rats that exhibited the highest rates of escalation. This highlights the importance of collecting data at multiple time points to determine the direction of causality between circuit strength differences and cocaine use.

Somewhat surprisingly, in the current study, there were similar changes in the arrangement of functional connectivity immediately after the cessation of self-administration in both cocaine and sucrose rats. While it was expected that cocaine self-administration would exclusively alter functional connectivity relative to sucrose self-administration, changes in the organization of functional connectivity after sucrose self-administration were not anticipated. There is, however, a precedent for sucrose inducing neurobiological changes that are similar to those resulting from the use of drugs of abuse (Westwater et al., 2016). For example, both cocaine and sugar consumption increase extracellular DA levels in the NAc (Di Chiara and Imperato, 1986, 1988; Rada et al., 2005). Additionally, the decreased striatal D_2_ receptor binding observed following chronic cocaine is also seen after sugar consumption (Colantuoni et al., 2001; Morgan et al., 2002; Spangler et al., 2004; Nader et al., 2006; Volkow et al., 2009). Many of the neurochemical changes that occur with sugar consumption only occur when sugar is available intermittently, which induces binge-like behavior; when sugar is available *ad libitum*, there are no changes in dopamine release or D_2_ receptor binding in the NAc (Spangler et al., 2004). In the current study, the sucrose control rats were yoked to the cocaine self-administration rats (such that their intake of sucrose was limited by the number of reinforcers obtained by their cocaine counterparts), which could be viewed as intermittent-like sugar intake. Thus, the similar changes in the arrangement of functional connectivity between cocaine and sucrose groups may be due to the fact that sucrose, a natural reinforcer, alters the same circuitry as cocaine, possibly resulting in an “addicted-like” state (Avena et al., 2014). Importantly, these changes in functional connectivity were absent in chamber exposure controls, indicating that the changes were specifically due to self-administration of cocaine or sucrose.

### Study limitations

With regard to the behavioral paradigm, although the number of reinforced responses was equalized between the cocaine and sucrose groups, there was still a difference in the total number of responses emitted as well as the time they spent in operant chambers. Whether this contributed to the differences in clustering coefficient following the 14 d self-administration sessions for both sucrose and cocaine remains unclear. Related to this, sucrose was used in the shaping of the operant responding for both sucrose and cocaine rats, and the exposure by both groups to sucrose could also have influenced the patterns of brain functional connectivity (which were increased across ROIs in both sucrose and cocaine groups). Nonetheless, rats in these groups exerted significant instrumental responses for each reinforcer, which were absent in the chamber exposure control group. This design was therefore critical to the present work.

In terms of the functional connectivity results, it should be noted that the measures employed in the current study cannot be used to determine the directionality of connectivity between brain regions, nor do they indicate that there are direct anatomic connections between nodes. The use of effective functional connectivity approaches such as Granger causality (Wen et al., 2013) or the use of more circuit-selective techniques such as optogenetics and tract tracing are better equipped to address these types of questions. Related to this, the significant overlap in the connectivity patterns observed in cocaine and sucrose rats could reflect the relatively low resolution of fMRI, which limits the resolution of potentially intermixed cocaine- or sucrose-specific circuits at a finer scale. In addition, as mentioned earlier, this study did not assess resting-state functional connectivity at longer abstinence time points. If, for instance, rats were imaged at a 30 d time point, other changes in node strength and connectivity might have been revealed. Indeed, some cocaine-induced neural alterations arise only well into abstinence (Christian et al., 2016). Thus, future studies will include resting-state functional connectivity measures at later time points, as this will provide a more thorough understanding of system-level changes following substance use. Finally, there were other limitations that should be noted. First, only males were included in the present work. Future studies should consider the effect of sex on functional connectivity networks affected by chronic cocaine use, as this is a highly relevant clinical question that needs to be addressed (Potenza et al., 2012). With regard to the imaging technique, the use of anesthetics, even if optimized for consistent measurement of resting-state networks (Lu et al., 2012), is a significant limitation when drawing comparisons with human neuroimaging studies of cocaine use disorders.

### Conclusion

In summary, these data demonstrate that both cocaine and sucrose self-administration can alter resting-state functional connectivity in brain regions involved in reward and affective processing, as well as those involved in attention and autonomic function. This study also highlights the importance of having multiple time points of comparison, such as a pretreatment baseline, to be able to explicitly dissociate pre-existing vulnerabilities from causal effects of substance use. Future work will examine whether these cocaine-induced changes in node strength and functional connectivity are unique to this stimulant, and how polysubstance use affects these same regions and circuits.

## Synthesis

Reviewing Editor: Karen Szumlinski, University of California at Santa Barbara Decisions are customarily a result of the Reviewing Editor and the peer reviewers coming together and discussing their recommendations until a consensus is reached. When revisions are invited, a fact-based synthesis statement explaining their decision and outlining what is needed to prepare a revision will be listed below. The following reviewer(s) agreed to reveal their identity: Rita Fuchs, Linda Porrino.

Both reviewers and I were very impressed by the efforts of the authors in revising this report for eNeuro. All three of us deem this report Acceptable As Is with no further comments or corrections at this time. Congratulations.
